# Myofilament-based physiological regulatory compensation preserves diastolic function in failing hearts with severe Ca^2+^ handling deficits

**DOI:** 10.1172/jci.insight.163334

**Published:** 2024-02-08

**Authors:** Frazer I. Heinis, Brian R. Thompson, Rishi Gulati, Matthew Wheelwright, Joseph M. Metzger

**Affiliations:** Department of Integrative Biology and Physiology, University of Minnesota Medical School, Minneapolis, Minnesota, USA.

**Keywords:** Cardiology, Calcium, Heart failure

## Abstract

Severe dysfunction in cardiac muscle intracellular Ca^2+^ handling is a common pathway underlying heart failure. Here we used an inducible genetic model of severe Ca^2+^ cycling dysfunction by the targeted temporal gene ablation of the cardiac Ca^2+^ ATPase, SERCA2, in otherwise normal adult mice. In this model, in vivo heart performance was minimally affected initially, even though Serca2a protein was markedly reduced. The mechanism underlying the sustained in vivo heart performance in the weeks prior to complete heart pump failure and death is not clear and is important to understand. Studies were primarily focused on understanding how in vivo diastolic function could be relatively normal under conditions of marked Serca2a deficiency. Interestingly, data show increased cardiac troponin I (cTnI) serine 23/24 phosphorylation content in hearts upon Serca2a ablation in vivo. We report that hearts isolated from the Serca2-deficient mice retained near normal heart pump functional responses to β-adrenergic stimulation. Unexpectedly, using genetic complementation models, in concert with inducible Serca2 ablation, data show that Serca2a-deficient hearts that also lacked the central β-adrenergic signaling–dependent Serca2a negative regulator, phospholamban (PLN), had severe diastolic dysfunction that could still be corrected by β-adrenergic stimulation. Notably, integrating a serines 23/24–to–alanine PKA-refractory sarcomere incorporated cTnI molecular switch complex in mice deficient in Serca2 showed blunting of β-adrenergic stimulation–mediated enhanced diastolic heart performance. Taken together, these data provide evidence of a compensatory regulatory role of the myofilaments as a critical physiological bridging mechanism to aid in preserving heart diastolic performance in failing hearts with severe Ca^2+^ handling deficits.

## Abstract 2

In failing hearts with severe calcium handling deficits, myofilaments can provide a bridging mechanism to aid in preserving diastolic performance.

## Introduction

Heart failure is a major cause of combined morbidity and mortality in humans worldwide (1–3). Despite best practices in clinical therapy, the 5-year mortality rate is very poor. Heart failure is a progressive and largely intractable clinical syndrome characterized by an overall decline in the pumping function of the heart. Heart failure can be subdivided clinically into heart failure with persevered ejection fraction (HFpEF) and heart failure with reduced ejection fraction (HFrEF) (4–6). HFpEF is just as common as HFrEF and results from poor myocardial muscle relaxation leading to inadequate filling and increased pressures in the left ventricular (LV) chamber, indicating marked diastolic dysfunction (7). HFpEF is prevalent in the aging population, wherein 40% or more of patients with heart failure have normal systolic performance yet the heart is failing (8). These patients present with the classic heart failure symptoms of breathlessness and fatigue owing to poor relaxation function of the LV chamber. There is no cure for heart failure except heart transplantation, which is woefully inadequate for the millions of patients with heart failure. Thus, there is an urgent need to discover and implement new strategies to aid the failing heart.

The molecular and cellular pathways to heart failure are complex and multifold (4). Of note, defective cardiac myocyte intracellular Ca^2+^ handling is a well-documented key component underlying diminished function in the failing heart (9). In this context, significant experimental focus has centered on a deeper mechanistic understanding of intracellular Ca^2+^ mishandling in the failing myocardium. In failure, transcription levels and protein content for the cardiac Ca^2+^ ATPase (SERCA2a), the key transporter of Ca^2+^ into the sarcoplasmic reticulum (SR), are significantly depressed in failing myocardial tissue as derived from animal studies and demonstrated in patients with heart failure (9, 10). A strong relationship follows, pointing to defective SR Ca^2+^ uptake and the consequent slow myocardial relaxation as a key component underlying diastolic heart failure.

The molecular complexities underlying heart failure make it challenging to implement animal heart failure models with defective SR Ca^2+^ uptake in the absence of other factors known to affect diastolic performance, including fibrosis, ischemia, and hypertrophy (4). In this context, a genetic system of inducible deletion of the cardiac SERCA2a gene provides an excellent model for investigating the relationship between impaired Ca^2+^ cycling and contractile dysfunction during heart failure, without complications due to structural alterations or ischemia (11–13). With high temporal and spatial precision, cardiac Serca2 gene deletion provides a unique model to efficiently delete Serca2a and ultimately leading to overt heart failure (11–15).

This model system has been studied extensively and is very well characterized from the myocyte to whole-organ levels (11–13, 15–23). Briefly, as overviewed here, upon Serca2 gene excision, SERCA2a protein content in the SR declines precipitously (half-life, 2.5 days) so that, from 2 weeks after ablation, there is little to no SERCA2a protein present (11–13). Most surprisingly, in vivo heart performance is little affected during the first 6 weeks after SERCA2a depletion, and only after that time does overt heart failure become apparent with death at approximately 8–10 weeks. This finding has been reported several times previously (13, 23). Loss of SERCA2a does not alter myocyte or ventricular morphology (no evidence of hypertrophy), does not cause structural alterations, and there is no evidence of fibrosis (13). Marked compensatory adaptations are notable in this model that preserve LV function, including marked increases in the trans-sarcomlemmal L-type Ca^2+^ current, increases in the L-type Ca^2+^ channel, and increases in the sodium/calcium exchanger (NCX) and in the plasmalemma ATPase (12, 13, 18–20, 23). This shifts the profile of Ca^2+^ cycling away from SR centered (95%) to downregulation in SR function with increased sarcolemma-based Ca^2+^ extrusion. This transition away from nearly exclusive SR-dependent intracellular Ca^2+^ handling in the mouse heart resembles, at least initially, a Ca^2+^ handling profile more like that observed in the human heart, where transsarcolemmal Ca^2+^ extrusion processes play a more substantial role than that in the normal mouse heart (13, 20, 24). Accordingly, hearts from the SERCA2-deficient mice are highly arrhythmia resistant, owing to the massive reduction in SR Ca^2+^ load (16). Isolated myocytes show marked reduction in twitch force and Ca^2+^ amplitude, and they show slowed Ca^2+^ transient decay rates (13). In vivo, serum catecholamines are elevated and the cAMP/PKA phosphoprotein content is correspondingly increased, including increased PKA-dependent phospholamban (PLN) phosphorylation (18). We, and others, hypothesize that these adaptations, including the heightened activation of cAMP/PKA phosphoprotein system (13, 18), are key in sustaining Ca^2+^ cycling and heart performance for several weeks in these mice after SERCA2 gene ablation (13, 18).

Therefore, this animal model serves as a unique system to investigate the myocardium-intrinsic mechanisms of diastolic performance under conditions of severe SR-based Ca^2+^ cycling deficits that are highly associated with the failing heart. Diastolic performance refers to the relaxation function of the heart muscle that, in turn, is dependent upon a complex interplay between myocyte intrinsic factors (Ca^2+^ cycling/myofilaments) and cell extrinsic factors including the extracellular matrix, fibrosis, and scarring. Thus, in this model of heart failure, the absence of LV remodeling, hypertrophy, fibrosis, or ischemia enables unique focus on myocyte intrinsic factors that affect mechanical relaxation performance in the transition to failure. The observation or near normal in vivo diastolic function, despite severe SR deficiency, suggests that myofilament mechanisms provide a functional bridge to abrogate impending myocardial failure.

We report here, in Serca2a-deficient hearts, β-adrenergic stimulation corrects contractile impairments at the level of the whole heart. Unexpectedly, using genetic complementation models, in concert with inducible Serca2 ablation, data show that Serca2a-deficient hearts that also lacked the key β-adrenergic signaling–dependent Serca2a negative regulator, PLN, had severe diastolic dysfunction that could still be corrected by β-adrenergic stimulation. This result pointed to β-adrenergic regulatory systems outside the canonical SR-based Ca^2+^ cycling pathway. Importantly, by using a sarcomere-incorporated PKA-refractory cardiac troponin I (TnI) molecular switch complex, results show a blunting of β-adrenergic stimulation–mediated enhanced heart relaxation performance. Collectively, these data provide evidence of an emerging compensatory regulatory role of the myofilaments as a physiological bridging mechanism to aid in preserving heart diastolic performance in failing hearts with severe Ca^2+^ handling deficits.

## Results

**Figure 1:**
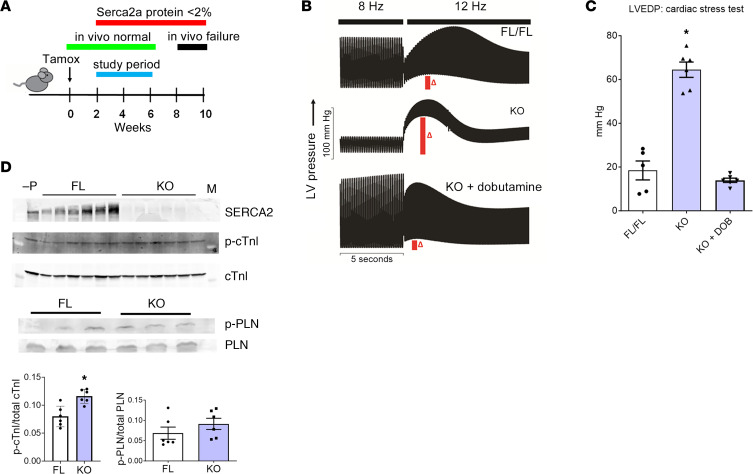
Marked restoration in LV function in SERCA2-KO hearts by acute β-adrenergic stimulation. (**A**) Timeline for studying LV performance in hearts isolated post Serca2 gene ablation, with experimental focus at 2–6 weeks, wherein Serca2a is depleted yet in vivo heart function is little affected. (**B**) SERCA2-KO and control FL hearts were evaluated by a stepped pacing protocol at baseline and during perfusion with/without 250 nM dobutamine in which pacing was increased from 7 to 12 Hz at an interval of 1 Hz per 30 seconds. After the 12 Hz pacing step under baseline or dobutamine-perfused conditions, hearts were returned to 7 Hz for 60 seconds, switched abruptly to 12 Hz for 30 seconds, and then returned to 7 Hz for washout. Vertical bars (red) with Δ indicate point at which summary data shown in **C** was collected. (**C**) Summary data of cardiac stress test–induced peak elevation in LV end-diastolic pressure (red vertical bars in **B**). One-way ANOVA with Bonferroni post hoc tests: *P < 0.05 versus FL/FL; ^#^*P* < 0.05 KO versus KO + DOB. FL, *n* = 9; KO, *n* = 6. (**D**) Western blots for SERCA2, p-cTnI, total cTnI, p-PLN, and total PLN. –P is a PLN KO control. Quantification of the phospho to total cTnI and PLN graphically represented. *n* = 6; *P = 0.0025.

A main focus of this work was centered on LV diastolic performance, as defined by mechanical relaxation of the ventricle, and were quantified here by reporting LV end-diastolic pressures (LVEDP) over a range of pacing (7–12 Hz) at the whole heart level (11) (Supplemental Figure 1; supplemental material available online with this article; https://doi.org/10.1172/jci.insight.163334DS1). LVEDP is a well-established clinical parameter of overall diastolic heart performance (25). Unless otherwise indicated, experiments were performed at 2–6 weeks after SERCA gene ablation (Figure 1A), a period during which it is well established that in vivo heart performance is surprisingly little affected, even though SERCA2a protein is very low (<2%) to undetectable (11, 13, 18–20). In terms of both residual SERCA2a content and LV function, data from animals between the 2- and 6-week after SERCA ablation period were comparable (schematized in Figure 1A). The majority of the data was collected at 4 weeks ± 1 week after ablation, with these data being similar. In these animals in vivo, data show that cardiac TnI (cTnI) serine 23/24 phosphorylation status is significantly increased in vivo (Figure 1).

### Severe myocardial diastolic dysfunction by inducible marked SERCA2a deficiency.

To further characterize the effects of SERCA2a deficiency on heart performance, Serca2^fl/fl^ mice engineered to express the αMHC-MerCreMer transgene (KO mice) were studied in comparison with non-MerCreMer transgenic Serca2^fl/fl^ littermates (FL mice), as controls. FL and KO mice were injected with tamoxifen to induce cardiac-specific Serca2 gene excision, and hearts were removed for ex vivo functional analysis by Langendorff methodology. Per the hypothesis guiding these studies, focus was on myocardial diastolic performance using LVEDP as a clinically meaningful parameter in relaxation function and the emergence of congestive heart failure (2, 4, 26). Time to 50% relaxation was also assessed as another valid parameter of heart diastolic function.

We, and several other groups, have well established highly efficient Serca2a gene excision with this inducible system as well as the consequent rapid loss of cardiac Serca2a protein content (Figure 1). As well documented in these previous studies, this model system enables the highly efficient and reproducible rapid decay in SERCA2a content, with an in vivo half-life of about 2.5 days (11–13, 18, 20, 22, 23). These published data establish that, from 2 weeks and onward after gene excision, SERCA2a protein content is at residual to undetectable levels (<2%; Figure 1) (11). These works further demonstrate that SERCA2a excision does not alter the content of other key SR/Ca^2+^ handling proteins, including no effect on RYR2, RYR-P, PLN, or calsequestrin protein content, nor is there any evidence of fibrosis or LV hypertrophy in these hearts (12, 13, 22).

In supplement to the main focus on mice studied at 4 weeks ± 1 week after SERCA gene excision (Figure 1A), in a separate study, we examined heart function at 1 and 2 weeks after tamoxifen, wherein FL and KO hearts were mounted in Langendorff mode and equilibrated at 7 Hz stimulation frequency for 10 minutes (Supplemental Figure 1). Hearts were then paced from 5 to 12 Hz in 1 Hz steps in a heart stress test pacing protocol well demonstrated to reveal diastolic dysfunction in disease models. At 1 week after excision, where Serca2a protein content is reduced by > 75%, LVEDPs were significantly increased during cardiac pacing. LVEDPs were further markedly increased in KO hearts during pacing stress at 2 weeks after excision, and LVEDPs remained elevated throughout the 2–6 weeks study period, where Serca2a protein is ~2% of control values (11–13). LVDPs were also significantly depressed at baseline pacing conditions (Supplemental Figure 1).

### Severe diastolic dysfunction in SERCA2a-deficient hearts corrected by β-adrenergic stimulation.

We next tested the effects of β-adrenergic stimulation on LV function in Serca2-KO hearts over the 2–6 weeks after gene excision period (Figure 1 and Supplemental Figure 1). For these studies using β-adrenergic agonists, we found similar acute results on LV function using dobutamine (DOB) and isoproteronol; thus, they are used here interchangeably in the following studies. Following pacing challenge, hearts were reequilibrated at 7 Hz for 10 minutes before perfusion was switched to Krebs containing 250 nM DOB, a β_1_ adrenergic agonist, for 15 minutes followed by a 10-minute washout with normal Krebs solution. Here, as above, KO hearts had marked systolic and diastolic impairment owning to Serca2 deficiency. Surprisingly, upon β-adrenergic agonist perfusion, both floxed controls and Serca2-KO hearts displayed similar (not statistically different) enhanced LVDP. Most notably, LVEDP, which was markedly increased in KO hearts, was fully corrected with LVEDP restored to control values upon β-adrenergic stimulation (Figure 1). Serca2 KO hearts also had a significant response to β-adrenergic stimulation in terms of ± dP/dt and T50Rise/T50Relax times (Supplemental Figure 2). These findings indicate a robust intact physiological response to β-adrenergic stimulation in terms of restoration of diastolic function, despite the marked Serca2 deficiency in the KO hearts.

### Chemical inhibition of SERCA2 function in KO hearts confirms SERCA2a deficiency.

**Figure 2:**
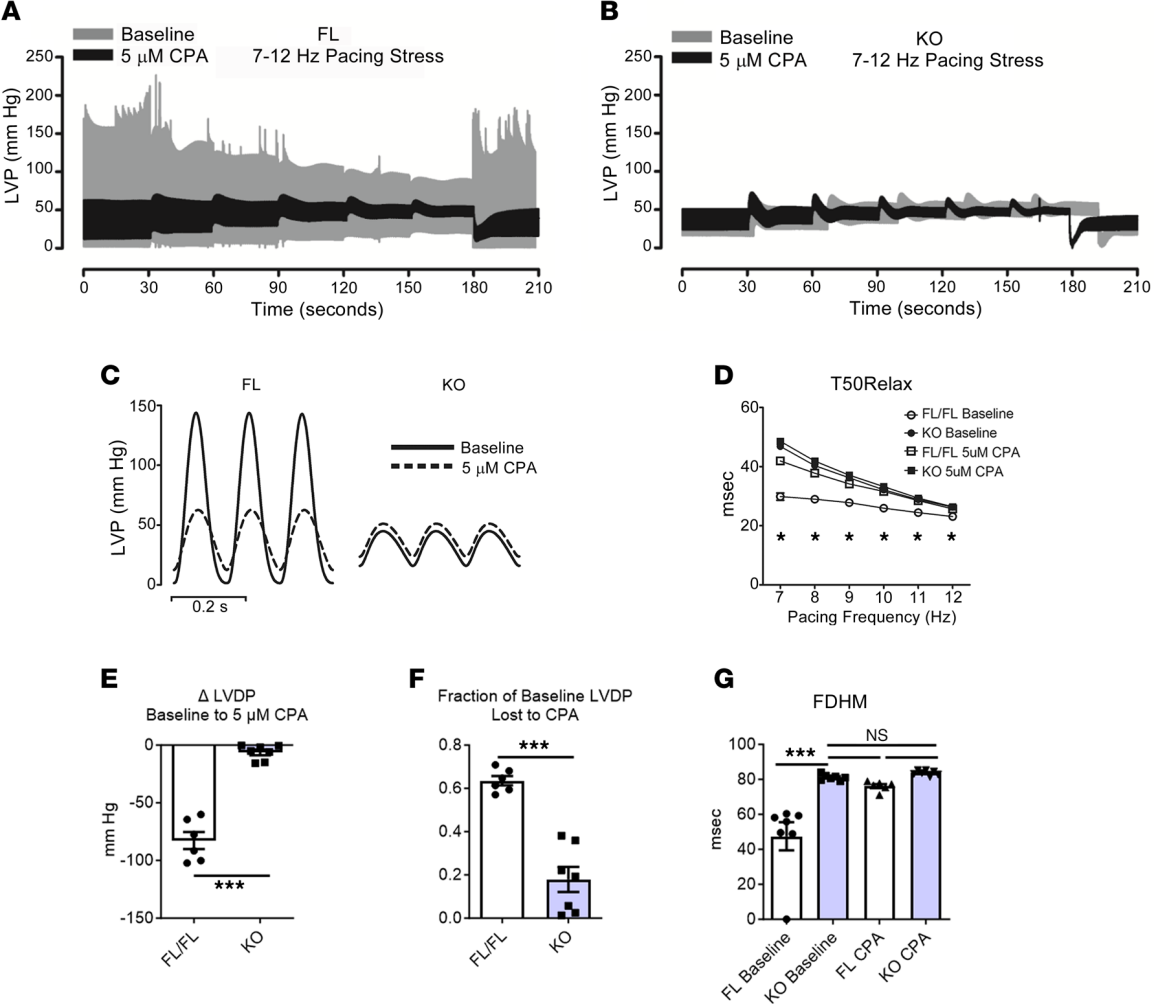
Effects of CPA chemical inhibition of the Serca2a ATPase in hearts after Serca2 gene ablation. KO data are at 2–6 weeks after gene ablation (Figure 1A). (**A** and **B**) Representative LV pressure traces from FL (**A**) and KO (**B**) hearts perfused with Krebs (gray trace) and 5 μM CPA (black trace). In each experiment, pacing was increased stepwise from 7 Hz to 12 Hz and then back to baseline 7 Hz, as in Supplemental Figure 2. (**C**) Representative traces of LV pressure from FL and SERCA2-KO hearts perfused with Krebs (solid line) and 5 μM CPA (dashed line). (**D**) Time from peak pressure to 50% decay during pacing steps from 7 to 12 Hz in FL and KO hearts. T50Relax was increased in FL hearts following CPA perfusion, but it was not significantly changed in KO hearts. Groups compared using 2-way ANOVA with Bonferroni post hoc tests. (**E**) Summary of change (Δ) in LVDP between baseline Krebs and 5 μM CPA perfusion. ***P < 0.0001 by 2-tailed Student’s *t* test. (**F**) Change in LVDP between baseline and CPA perfusion expressed as a fraction of baseline LVDP. ***P < 0.0001 by 2-tailed Student’s *t* test. (**G**) Full-duration at half-maximum (FDHM), the sum of T50Rise and T50Relax representing the duration of the active state of contraction, for FL and KO hearts at 7 Hz. FDHM of KO hearts is not affected by CPA treatment. *n* = 6 FL, 7 KO.

Owing to the fast turnover of SERCA2a protein in the heart, t_1/2_ 2.5 days, we and others find residual to undetectable SERCA2a protein starting a 2 weeks and beyond after gene excision (Figure 1D) (11, 13, 22, 23). Nonetheless, we investigated whether any residual SERCA2 protein could help account for the robust dynamic physiological performance we observed in the SERCA2-KO hearts upon β-adrenergic stimulation (Figure 2). Thus, FL and KO hearts were perfused with the potent SERCA2 inhibitor, cyclopiazonic acid (CPA, 5 μM in Krebs). Upon CPA perfusion in FL hearts, LVDP markedly decreased and LVEDP significantly increased (Figure 2). In contrast, CPA had little effect to no effect on the SERCA-KO hearts. Here, upon cardiac pacing stress, end-diastolic pressures increased to a greater degree in FL hearts than in SERCA2 KO hearts. T50Rise and T50Relax were significantly increased in FL hearts following CPA perfusion but were not significantly altered by CPA in KO hearts (Supplemental Figure 3). The LV pressure full-duration at half maximum (FDHM, the sum of T50Rise and T50Relax) was significantly increased with CPA treatment in FL controls, whereas it had no significant effect in KO hearts (Figure 2 and Supplemental Figure 3).

Next, after perfusion with 5 μM CPA, FL and KO hearts were then perfused with Krebs containing both 5 μM CPA and 50 nM isoproterenol (ISO) to test for any residual β-adrenergic reserve in the context of serve SERCA2 deficiency together with acute SERCA2 chemical inhibition. As above, with CPA, T50Rise and T50Relax significantly increased in FL hearts, with no effect detected in KO hearts, between baseline and 5 μM CPA (Supplemental Figure 3). Interestingly, T50Rise and T50Relax were significantly and comparably altered in FL and KO hearts when ISO was added during CPA SERCA2 inhibition (Supplemental Figure 3). Overall, these data provide additional support for the lack of functional SR in these hearts.

### High-Ca^2+^ inotropy does not correct relaxation deficits of KO hearts.

**Figure 3:**
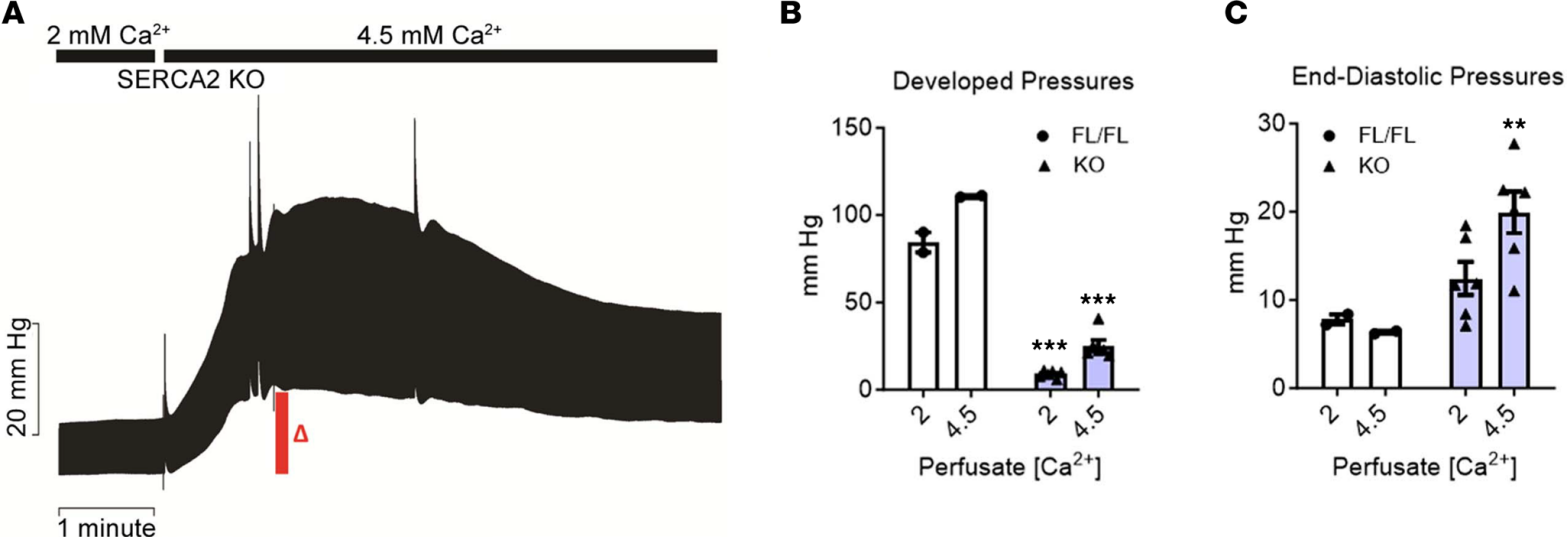
Increased LV developed pressure by high calcium in perfusate does not correct increased diastolic pressures in SERCA2-KO hearts. (**A**) Representative original LV pressure traces from SERCA2-KO heart as it is switched from Krebs containing 2.5 mM Ca^2+^ and 0.5 mM EDTA to Krebs containing 5 mM Ca^2+^ and 0.5 mM EDTA. (**B** and **C**) LVDP (**B**) and LVEDP (**C**) for FL (*n* = 2) and 4-week KO (*n* = 6) hearts under normal and high-Ca Krebs. KO 4.5 mM versus 2.5 mM: **P < 0.01, ***P < 0.001.

Diastolic performance is known to be sensitive to mechanical effects stemming from the magnitude of systolic pressure development, as attributed to the physiological process of dynamic recoil on myocardial relaxation (27–30). Thus, it was unclear whether the improved relaxation function observed in KO hearts upon β-adrenergic stimulation was due to dynamic recoil effects from the increased systolic performance or rather was due to mechanical load–independent myocyte cell intrinsic–dependent processes. To investigate this, systolic function was directly increased by increasing the [Ca^2+^] in the perfusate. FL and KO hearts perfused with high [Ca^2+^] had similar magnitude increases in systolic pressures, as compared with baseline perfusion (Figure 3). Interestingly, whereas LVEDP was not significantly altered in FL under high Ca^2+^, end-diastolic pressures were markedly increased in KO hearts by high-perfusate Ca^2+^ (Figure 3). These data are evidence that the β-adrenergic stimulation–mediated enhanced relaxation performance in SERCA-KO hearts is not due to the increased systolic performance-based elastic recoil, or related mechanical effects, but rather indicates that myocyte intrinsic signaling processes underlie the enhanced diastolic performance.

### Genetic dissection of relaxation performance in SERCA2-deficient hearts regarding the role of PLN.

**Figure 4:**
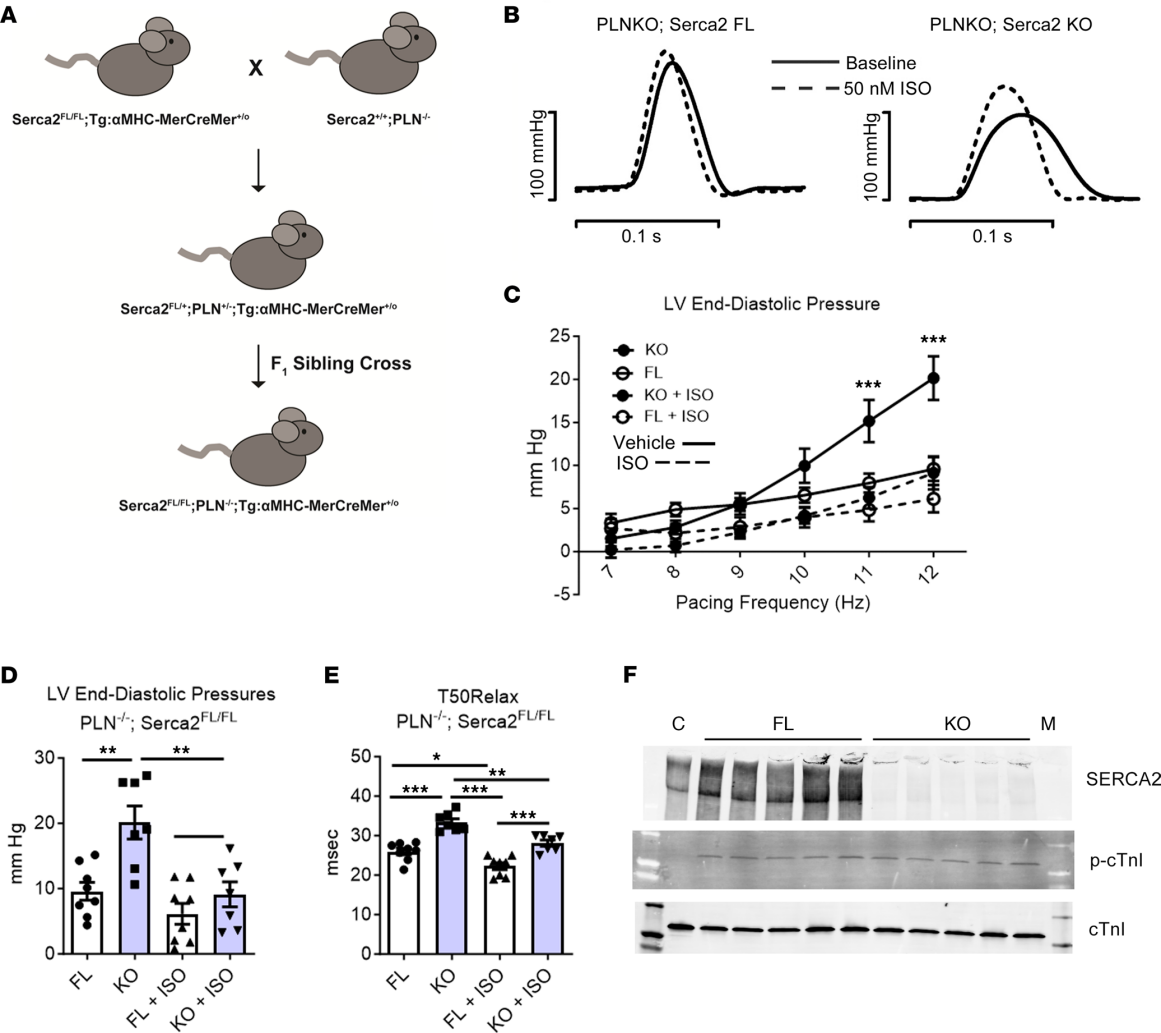
Effects of PLN gene KO on LV performance in Serca2 gene–ablated hearts. (**A**) SERCA2-KO × PLN-KO breeding diagram. Serca2^fl/fl^ mice were crossed with PLN^–/–^ (PLN-KO) mice lacking the key SERCA2 inhibitor, phospholamban (PLN). F1 heterozygous siblings were crossed, and the Serca2-FL and PLN-KO alleles were bred to homozygosity. These mice either expressed (TG) or did not express (NTG) the αMHC-MerCreMer transgene allowing for Serca2 gene disruption upon tamoxifen injection. For experiments using these mice, all animals lacked PLN and were either Serca2^fl/fl^ (PLN-KO;Serca2-FL) or Serca2-KO (PLN-KO;Serca2-KO). (**B**–**D**) Serca2^fl/fl^;PLN^–/–^ mice were injected with tamoxifen (40 mg/kg i.p. in peanut oil) to induce Serca2 KO in mice expressing MerCreMer recombinase. Hearts were excised from SERCA2-FL or SERCA2-KO mice and evaluated by Langendorff perfusion. Hearts were equilibrated for 15 minutes in normal Krebs (vehicle), paced from 7 to 12 Hz in 1 Hz increments, and reequilibrated for 10 minutes at 7 Hz before switching perfusion to a reservoir containing 50 nM isoproterenol in Krebs. The 7–12 Hz pacing challenge was repeated, and hearts were returned to normal Krebs at 7 Hz for 10 minutes. (**B**) Representative original LV pressure recordings in Serca2 FL and KO hearts with complete PLN deficiency and in the presence or absence of acute β-adrenergic stimulation. (**C**) LVEDP during pacing challenge. At baseline, end-diastolic pressures in FL hearts do not rise above preload levels, whereas KO hearts are unable to relax fully at high pacing frequencies. After perfusion with 50 nM isoproterenol, KO hearts relax fully at all pacing frequencies, indicating an intact lusitropic effect of isoproterenol in a PLN^–/–^ heart. ***P < 0.05 for KO (no Iso) versus all other groups at that pacing frequency. (**D**) LVEDP at maximum pacing frequency in SERCA2-KO; PLN^–/–^ hearts. Serca2-KO; PLN^–/–^ hearts are unable to relax fully at high pacing frequencies. This impaired relaxation performance is corrected upon isoproterenol perfusion. Groups were compared using 1-way ANOVA with Bonferroni post hoc tests. **P < 0.01. *n* = 9 per group. (**E**) T50 Relax at 7Hz ***P < 0.001, **P = 00.012, *P = 0.0171 using 1-way ANOVA with Tukey post hoc test. (**F**) Western blots for SERCA2, p-cTnI, and total cTnI in PLN-KO hearts. C, a control SERCA replete heart sample; M, Marker. *n* = 5 per group.

**Figure 5:**
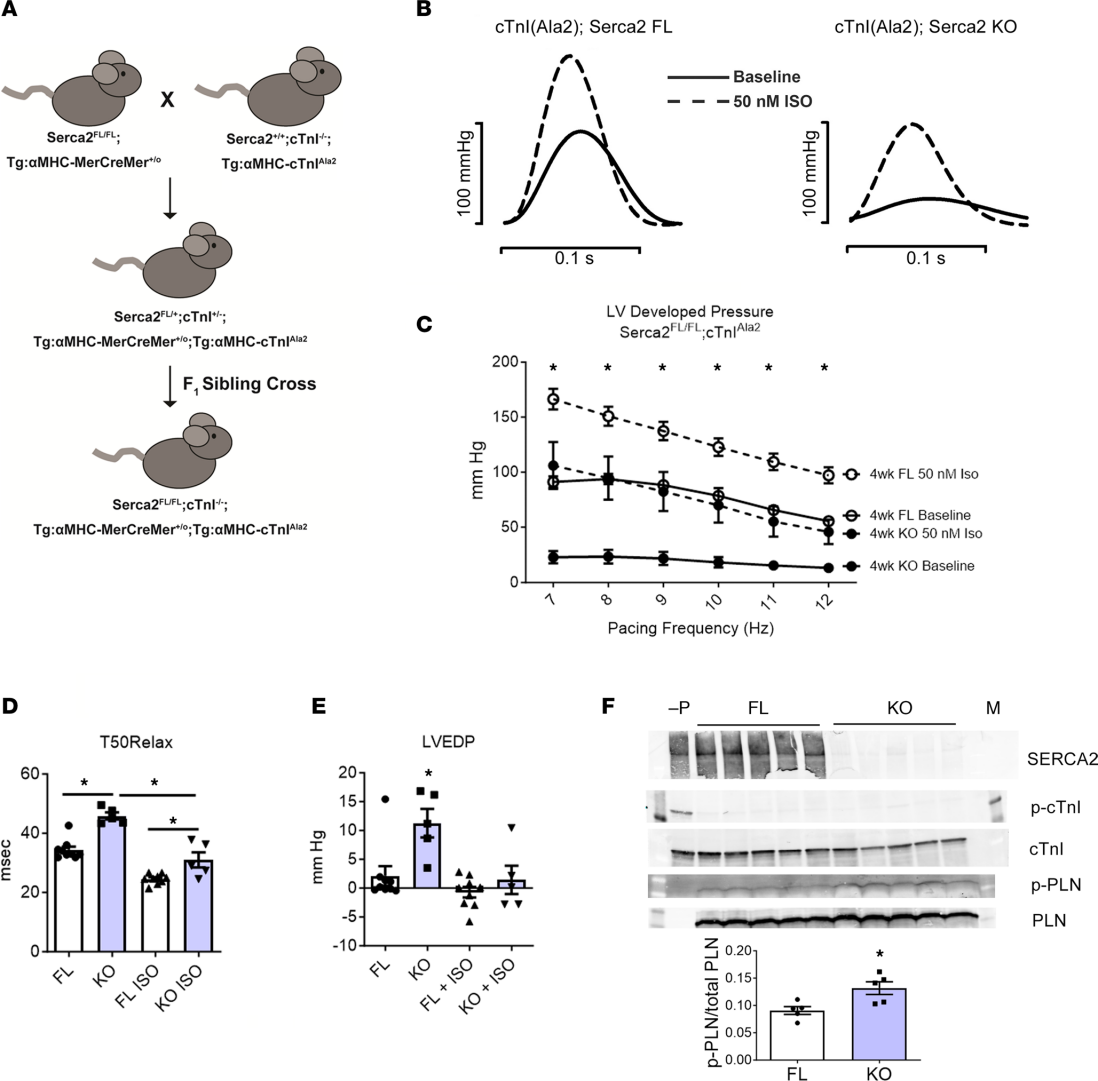
Effects of myofilament incorporation of cTnI(Ala2) on LV performance in Serca2 gene–ablated hearts. (**A**) Breeding scheme. Serca2^fl/fl^ × cTnIAla2 breeding scheme. Serca2^fl/fl^;Tg:αMHC-MerCreMer mice were bred with mice expressing a PKA-insensitive mutant of cardiac troponin I, cTnIAla2, in which the PKA target serines 23/24 have been mutated to alanine. To ensure complete expression of the PKA-insensitive transgene, this line is maintained as cTnI^–/–^;Tg:αMHC-cTnIAla2. Heterozygous siblings were bred to homozygosity to create the mouse line Serca2^fl/fl^; cTnI^–/–^;Tg:αMHC-MerCreMer;Tg:αMHC-cTnIAla2, in which PKA phosphorylation of cTnI cannot contribute to the lusitropic response upon β-adrenergic stimulation. (**B**) Representative original LV pressure recordings in Serca2-FL and -KO hearts with complete cTnI(Ala2) incorporation into the myofilaments and studied in the presence or absence of acute β-adrenergic stimulation. (**C**) LV end-diastolic pressures of FL and SERCA2-KO hearts expressing cTnIAla2. Isoproterenol perfusion reduces LVEDP in both groups, but SERCA2-KO hearts are unable to completely relax at high-pacing frequencies. FL (*n* = 9) and SERCA2-KO (*n* = 5) hearts all expressed PKA-insensitive cTnIAla2, during pacing challenge. (**D**) T50Relax times during pacing challenge of FL and KO hearts expressing cTnIAla2. Isoproterenol perfusion decreases T50Relax in both FL and KO groups. KO hearts perfused with isoproterenol remain significantly slower than isoproterenol-perfused FL hearts. Two-way ANOVA with Bonferroni post hoc tests: FL + ISO versus KO + ISO tests are *P* < 0.01 or lower for all pacing frequencies except 12 Hz (*P* > 0.05). (**E**) LVEDP as a measure of relaxation is reduced with ISO. *P < 0.05. (**F**) Western blots of SERCA2, p-cTnI, total cTnI, p-PLN, and total PLN. –P, PLN-KO control; M, marker. Quantification of p-PLN to total PLN content graphically represented. *P = 0.0166, *n* = 5 per group.

An unexpected finding here is that the SERCA2-KO hearts retained a highly significant diastolic functional reserve upon β-adrenergic stimulation and not different from that of control SERCA2 replete hearts. β-Adrenergic stimulation is well known to markedly enhance myocardial contractile performance via several key phosphoproteins. In terms of relaxation performance, a prominent and well-studied phosphoprotein is PLN (9). Thus, to investigate the mechanism supporting intact β-adrenergic responses in SERCA2-depleted hearts, Serca2^fl/fl^ mice were crossbred with PLN^–/–^ mice to probe the interplay between adrenergic signaling and PLN-based Ca^2+^ cycling in the SERCA2a-deficient heart. The Serca2^fl/fl^;PLN^–/–^ mouse line was generated using F1 heterozygous siblings crossed and the Serca2-FL and PLN-KO (PLN^–/–^) alleles were bred to homozygosity (Figure 4). These mice either expressed (TG) or did not express (NTG) the αMHC-MerCreMer transgene, allowing for Serca2 gene disruption upon tamoxifen injection. For experiments using these mice, all animals lacked PLN and were either Serca2*^fl/fl^* (PLN-KO;Serca2-FL) or Serca2-KO (PLN-KO;Serca2-KO). Serca2 and PLN ablation are confirmed in Figure 4 and Figure 5. At baseline, PLN-KO;Serca2-KO hearts had significantly lower LV-developed pressures than PLN-KO;Serca2-FL hearts (Supplemental Figure 4). During cardiac stress testing at high pacing frequencies, PLN-KO;Serca2-KO hearts had significantly elevated LVEDP, as compared with PLN-KO;Serca2-FL hearts (Figure 4). β-Adrenergic stimulation had no effect on systolic or diastolic performance in PLN-KO;Serca2-FL hearts, as reported earlier in PLN-KO hearts (9). Surprisingly, β-adrenergic stimulation significantly improved LVDP and LVEDP in the PLN-KO;Serca2-KO hearts (Figure 4 and Supplemental Figure 4). In addition, β-adrenergic stimulation significantly decreased T50Rise and T50Relax times in PLN-KO;Serca2-KO hearts. In contrast, T50Rise and T50Relax times were not altered by β-adrenergic stimulation in PLN-KO;Serca2-FL hearts (Supplemental Figure 4).

### Genetic dissection of relaxation performance in SERCA2-deficient hearts regarding the role of myofilaments.

Based on the unexpected finding that β-adrenergic stimulation markedly enhanced myocardial relaxation in hearts depleted of both SERCA2 and PLN, we next investigated the role of myofilaments. We focused on cTnI, a well-studied phosphoprotein in the myofilaments known to have a modulatory role in myocardial relaxation performance in response to β-adrenergic stimulation (31, 32). To this end, a new mouse model was developed wherein Serca2*^fl/fl^*;Tg:αMHC-MerCreMer mice were crossed with mice expressing a PKA-insensitive mutant allele of cTnI, cTnI^Ala2^, in which all the PKA target serines 23/24 have been mutated to alanines (Figure 5). To ensure the complete expression of the PKA-insensitive cTnI transgene in the sarcomeres, this line was maintained on the homozygous cTnI-null background: cTnI^–/–^;Tg:αMHC-cTnI^Ala2^. To this end, heterozygous siblings were bred to homozygosity to generate the mouse line Serca2*^fl/fl^*;cTnI^–/–^;Tg:αMHC-MerCreMer;Tg:αMHC-cTnI^Ala2^. Thus, in this line all sarcomeric cTnI is cTnI^Ala2^ (33). Accordingly, in terms of cTnI, this line is fully refractory to PKA-based serine 23/24 phosphorylation, as confirmed here (Figure 5F).

Upon Serca2 gene excision, at baseline pacing conditions, the Serca2-KO;cTnI^Ala2^ hearts had greatly reduced systolic and diastolic function, as compared with FL controls (Figure 5). Developed pressures in FL hearts, and in ISO-perfused KO hearts, followed a negative staircase as pacing frequency increased (Figure 5). β-Adrenergic stimulation caused a significant decrease in LVEDP in both FL and KO groups. Interestingly, KO hearts could not relax as fast (T50Relax slower) as FL during β-adrenergic stimulation (Figure 5). These data provide evidence for a key role of cTnI as a physiologically relevant phosphoprotein in facilitating relaxation performance under conditions of severe SERCA2 deficiency.

### In vivo effects of β-adrenergic signaling blockade in SERCA2-KO mice.

**Figure 6:**
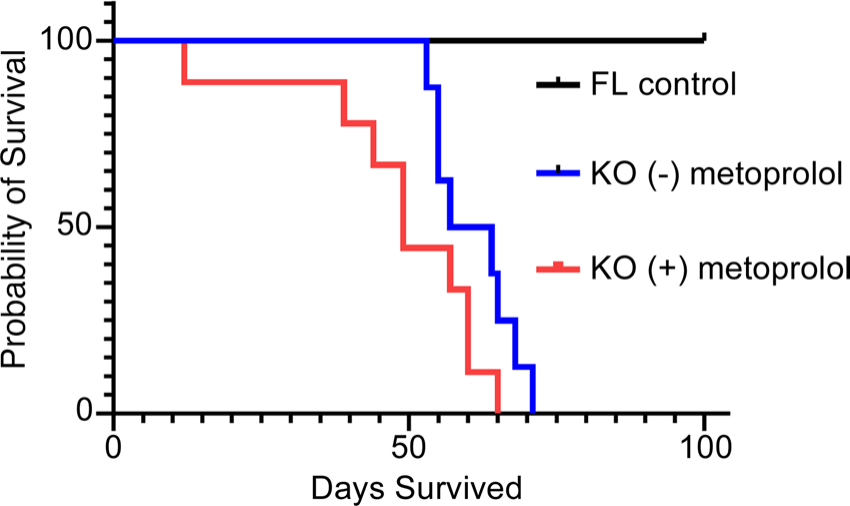
Survival study in mice after Serca2 gene ablation in the presence and absence of chronic β-adrenergic axis blockade. KO mice were provided drinking water with metoprolol (2 mg/mL) after tamoxifen injection (40 mg/kg i.p. in peanut oil). Two studies were performed with all data presented in the figure. The first group (metoprolol) contained 3 males and 6 females, and the second group (no metoprolol control) contained 5 males and 4 females. Both groups received a tamoxifen injection to induce excision of the SERCA2a gene by Cre recombinase and ablation of SERCA2a expression. They were given metoprolol, a selective β1-adrenergic receptor antagonist, at a concentration of 2 mg/mL in their drinking water from the time of tamoxifen injection until death.

It has been proposed previously that, under conditions of severe SERCA2a deficiency, physiological compensation may sustain heart performance via heightened sympatho-adrenal axis stimulation (18). This is supported by our finding of elevated cTnI serine 23/24 phosphorylation status in the heart in vivo (Figure 1). Thus, to test whether increases in β-adrenergic signaling following *Serca2* gene disruption may contribute to sustaining cardiac function in SERCA2-KO mice in vivo, KO mice were administered metoprolol, a β_AR_ inhibitor, in the drinking water (2 mg/mL) following tamoxifen administration. Overall, there was a significant survival difference among controls and the SERCA-excised mice, with or without metoprolol. Data further show that KO mice with metoprolol died earlier than the KO mice (–) metoprolol, with more than 50% dead prior to a single death in the (–) metoprolol group (Figure 6). KO mice given metoprolol survived for a median duration of 41.5 days, as compared with 59.5 days for KO mice given normal drinking water. However, this difference did not gain significance over the full survivor analysis (*P* = 0.065). Collectively, these data underscore the complexities of survival analysis in this model system. These results further suggest that multiple signaling mechanisms are likely present that contribute to animal survival after Serca2 ablation. Ultimately, the complete mechanistic dissection of these signaling processes will be challenging to dissect and will require extensive future experimentation to bring to resolution.

## Discussion

Heart failure is a complex clinical syndrome of reduced heart performance (4, 8, 34). Mechanistic studies of heart failure are challenging, owing to the multiple pathways and signaling networks affected (4, 25, 35). One unifying theme of the failing heart involves, at least in part, marked derangements in the intracellular Ca^2+^ handling processes controlling myocardial function (9). Whereas Ca^2+^ handling dysfunction is prominent in the failing human heart, dissecting its role in failure is complicated by the myriad alterations arising from other factors, including structural effects, hypertrophy, scarring/fibrosis, and ischemia. Here, we investigated the role of severe Ca^2+^ handling dysfunction in heart performance using a genetic model of inducible SERCA2 gene excision leading to failure without hypertrophy, fibrosis, or ischemia (11–15, 20, 22, 23).

Our main new findings show a compensatory increase in cTnI serine 23/24 phosphorylation content in hearts in vivo upon SERCA2a depletion. We propose that this serves as a physiologically relevant mechanism to enhance diastolic function under conditions of severe Ca^2+^ cycling dysfunction. Data show a robust heart pump functional reserve capacity in the SERCA2a-depleted hearts. Notably, upon β-adrenergic stimulation, marked deficits in diastolic performance in the SERCA2-KO hearts were fully corrected and not different than control SERCA2 replete hearts. Data further show complete β-adrenergic stimulation–mediated correction in diastolic performance in SERCA2a-deficient hearts in which PLN, the PKA-dependent dominant negative regulator of SERCA2a, was ablated. Importantly, transgenic hearts with sarcomeric incorporation of a PKA-insensitive cTnI molecule markedly blunted the effect of the β-adrenergic stimulation to correct diastolic performance in SERCA2a-deficient hearts. This result derives from a posttranslational regulatory effect of cTnI to titrate sarcomeric Ca^2+^ sensitivity as a mechanism to facilitate hastened myofilament relaxation (31, 32). Collectively, these findings provide mechanistic insights into the failing myocardium by unmasking a major role of dynamic compensatory myofilament regulatory performance in serving as a key physiological bridge to preserve diastolic function in hearts with severe Ca^2+^ handling deficits.

In humans, compensatory heightened activation of the β-adrenergic axis is a physiological bridging mechanism to stem heart pump failure resulting from severe Ca^2+^ handling dysregulation (4, 35). Serum catecholamines are also shown to be elevated in heart failure animal models with SERCA2a deficiency (18). In particular reference to diastolic performance, we found that severely SERCA2a-depleted hearts that also lack the key PKA-target phosphoprotein PLN maintain robust β-adrenergic stimulation–dependent enhanced myocardial relaxation performance. This finding is in contradistinction with present paradigms of essential and near exclusive control elements of SERCA2a/PLN in the dynamic control of diastolic heart performance (9). Our results suggest that the physiological interplay between the SR and myofilaments shifts dramatically toward an increased physiologically relevant role of the myofilaments governing relaxation performance under conditions with marked Ca^2+^ handling dysregulation. Thus, wherein healthy heart diastolic performance is dominated by SR control mechanisms (most notably in rodents), in failure, this shifts to the myofilaments. Because in the healthy human heart, Ca^2+^ handling control is significantly less SR dependent than in the murine system (36), we posit that the results provided here would translate physiologically to the failing human heart, pointing to an even greater role for myofilaments in regulatory control of diastolic performance in human myocardium than demonstrated here in the mouse model. This, in turn, suggests an opportunity to target the sarcomere for diastolic dysfunction remediation in the failing heart.

Our findings provide evidence that the mechanism by which compensatory modification of myofilaments serve to regulate myocardial relaxation performance during failure directly involves the molecular switch molecule of the sarcomere, cTnI. It is known that cTnI is a PKA-dependent phosphoprotein with capacity for dynamic modulatory control of myofilament responsiveness to cycling intracellular Ca^2+^ (31, 32, 37). Previously, we showed in transgenic mice and in direct gene transfer studies that myocytes engineered with the constitutively on PKA-mimetic cTnI serines 23/24D incorporated into the sarcomeres had a direct effect to accelerate myocyte relaxation (31). That study provided direct evidence that both cTnI and PLN are critical in modulating cardiac muscle relaxation performance. It was further postulated that, under disease conditions featuring Ca^2+^ mishandling, as in heart failure, cTnI would have a markedly greater role in modulating relaxation performance. The present findings provide direct support to this hypothesis by showing PKA-refractory cTnI serines 23/24A significantly blunting diastolic performance in hearts with SERCA2a deficiency. This is demonstrated by the significant difference in LVEDPs observed in β-adrenergic–stimulated Ala2 hearts, with and without Serca2. Because cTnI serines 23/24A significantly, but incompletely, abrogated β-adrenergic stimulation relaxation performance, other myofilament proteins are likely also involved in compensating for Ca^2+^ dysregulation–based diastolic function. In this context, several candidates can be considered, including MyBP-C, a sarcomeric phosphoprotein shown to modulate cardiac performance (37). Additional studies will be required to fully dissect any role of MyBP-C or other myofilament proteins in including notably TnT, myosin, titin, and others in further refining diastolic performance in the failing heart.

Numerous animal models have been developed to investigate the complex mechanisms underlying heart failure. These include ischemic injury, pressure overload, pacing, diet, and numerous genetic models (4, 38–40). Presently, there is no ideal heart failure model, as each has its own inherent strengths and limitations. This is not surprising, because, in human heart failure, it is understood that the pathways to pump failure are complex and multifold (4, 25, 35). That said, there is strong evidence that dysregulation in cardiac Ca^2+^ handling is a unifying component of the heart failure signature (9). With this in mind, we focused studies on otherwise normal mice that can be reproducibly induced to overt heart failure upon temporally regulated control of SERCA2 gene deletion (11–13, 18, 22, 23). A strength of the model rests in high precision and reproducibility of this system’s highly efficient gene excision mechanism coupled with the short half-life of SERCA2a in vivo (11–13). This model affords the opportunity to isolate effects due to Ca^2+^ handling deficits independently of other heart failure associated alterations, including fibrosis, mal-adaptive hypertrophy, and ischemia that have also been implicated in diastolic heart failure (4). In addition, the results here in a murine model of inducible heart failure find parallels in rabbit whole heart studies in which the Serca2a pump was disabled via chemical inhibition. Upon application of thapsigargin, and blockade of SR-based Ca^2+^ uptake, the rabbit heart performed at an unexpectedly high level for hours, prior to complete pump failure (41). This is important, as the intracellular Ca^2+^-handling process in the rabbit heart parallels closely that of the human myocardium (36).

It is further apparent that the molecular dissection of the relative roles of cTnI and PLN in β-adrenergic–mediated hastening of cardiac relaxation has been challenging and controversial. For example, as we previously discussed (31), the question can be advanced as to whether data from PLN-KO mice are informative in understanding the basis of relaxation in otherwise normal hearts with both PLN and TnI present. As we have discussed previously, in a converse experiment where TnI is ablated, force develops in the absence of altered intracellular calcium (31). These studies indicate the essential role of TnI in the heart; however, they cannot be taken as evidence of the relative importance of TnI and PLN as determinants of relaxation in the context of normal heart performance. Based on the present findings, TnI-P clearly has an essential role in the normal myocyte expressing both SERCA2a/PLN. Furthermore, PLN, as the key inhibitor of SERCA2 function, plays a dominant role in the context of the genetically engineered PLN-deficient (and TnI replete) heart that has unfettered SERCA2a function by virtue of PLN ablation (42). Thus, experiments performed in the absence of PLN, and with normal TnI, shift the relative importance of cTnI-PLN toward PLN, with unfettered SERCA2a-based Ca^2+^ uptake capacity.

Clinical treatment for human heart failure involves application of drugs that block the β-adrenergic stimulation pathway (43–45). β-Blockers have been shown to be effective in reducing morbidity and mortality in patients with heart failure. Nonetheless, despite excellent care, the 5-year survival rate for patients remains very poor. In discussing our findings in the context of heart failure clinical care, we speculate that β-blocker application is a double-edged sword, in terms of diastolic performance. On the one hand, the negative chronotropic effects of these drugs would serve to prolong diastole to help mitigate effects due to slow myocardial relaxation. However, myofilament-based compensatory effects would also be blunted and place physiological limits on beat-to-beat pacing necessary to preserve adequate chamber filling in diastole. One consequence of this treatment regime is the well-noted negative effects on exercise tolerance in patients with heart failure (4).

In summary, we have shown that hearts with severe SERCA2a deficiency and profound cardiac Ca^2+^ cycling impairment remain capable of robust functional responses to β-adrenergic stimulation. This is particularly striking, in terms of the preserved diastolic function, as Serca2a-based Ca^2+^ handling is thought to be the essential pathway required for physiological relaxation performance (9). Genetic dissection of canonical PKA-based regulatory phosphoproteins implicate cardiac troponin as having a key role in bridging physiological diastolic heart function under conditions of severe Ca^2+^ cycling dysfunction, as observed in the failing heart (9). These findings suggest a shift from SR-based to myofilament-based diastolic regulatory control process in Ca^2+^ handling deficient failing hearts. We posit that, if the regulatory lusitropic effects of cTnI, as evidenced in the present study, could be translated to the human heart, this may reduce the requirements for negative chronotropic acting drugs and, in turn, improve exercise tolerance and survival.

## Methods

### Sex as a biological variant

Groups were studied and analyzed in using blinded experimental design. Adult male and female mice (age 3–5 months) were studied with no clear differences discerned by sex; accordingly, the male and female results were pooled. All lines were backcrossed onto C57BL/6 genetic background.

### Animal handling

Mice were housed on a 12-12 hour light-dark cycle and provided rodent chow and tap water ad libitum. All mice were homozygous for loxP sites in introns 1 and 3 of the Serca2 gene (Serca2^fl/fl^), and either contained (TG) or did not contain (NTG) the αMHC-MerCreMer transgene (transgene was heterozygous in all groups). Mice provided by Geir Christensen (Ullevaal University Hospital, Oslo, Norway). All mice were genotyped, as described previously (11). All mice were injected with tamoxifen dissolved at 10 mg/mL in peanut oil (1× 40 mg/kg i.p.). As we and others show in this system, Serca2^fl/fl^;Tg:αMHC-MerCreMer^+/o^ mice efficiently deleted Serca2 in response to tamoxifen and became severely Serca2 protein deficient (KO) in the heart (11–13, 15, 17–23). Serca2^fl/fl^; NTG littermate animals serve as controls as they retained the floxed Serca2 gene and have normal SERCA2 expression (FL controls).

### Langendorff procedure

We used procedures well validated and implemented by us previously to assess mouse whole heart function (11, 46–50). Mice were anesthetized with sodium pentobarbital (100 mg/kg i.p.) and heparinized (250 IU i.p.). Upon loss of toe pinch reflex, the ribcage was opened and the heart was removed to a dish of ice-cold Krebs-Henseleit solution (KHB: [in mmol/L] 118 NaCl, 4.7 KCl, 1.2 MgSO_4_, 1.2 KH_2_PO_4_, 0.5 NaEDTA, 2.5 CaCl_2_, 10 glucose, 25 NaHCO_3_; Sigma-Aldrich). The aorta was trimmed and cannulated, and the heart was mounted on a Langendorff apparatus (Radnoti Inc.) and retrogradely perfused with KHB bubbled with 95% O_2_/5% CO_2_ and maintained near 37°C with a water jacket. The atria were removed, and a balloon catheter was inserted into the left ventricle (LV) to measure isovolumic LV pressure. An electrode was placed at the base of the heart controlled pacing frequency, which was set at 7 Hz for baseline and equilibration. Balloon preload was set between 5 and 10 mmHg. LVDPs were robust as consistent with a well-perfused healthy myocardium.

### Langendorff pacing and drug perfusion protocols

Isolated hearts were perfused with normal Krebs at 7 Hz for 15 minutes for initial equilibration, and then the pacing frequency was adjusted from 7 to 12 Hz in increments of 1 Hz per 30 seconds (11). After this pacing challenge, hearts were returned to 7 Hz for 10 minutes; then, perfusion was switched from Krebs to a reservoir containing Krebs and either 250 nM DOB, 50 nM ISO) as β-adrenergic agonists, or 5 μM CPA. In this study, the acute effects of DOB and ISO were similar. For DOB and ISO perfusion protocols, 10 minutes after switching perfusate, the 7–12 Hz pacing challenge was repeated; then, hearts were returned to normal Krebs at 7 Hz for 10 minutes and removed from the apparatus. For CPA protocols, 5 minutes after switching perfusate, the 7–12 Hz pacing challenge was repeated; then, hearts were reequilibrated at 7 Hz for 2 minutes. Perfusion was then switched to a third reservoir containing 5 μM CPA and 50 nM ISO for 5 minutes, 7–12 Hz pacing was repeated, and hearts were washed out in normal Krebs for at least 10 minutes. Hearts were blotted dry, frozen using liquid nitrogen, and stored at –80°C.

### Serca2fl/fl breeding

Serca2^fl/fl^ mice were crossed with PLN^–/–^ (PLN-KO) mice lacking the key SERCA2 inhibitor, PLN (42). F1 heterozygous siblings were crossed, and the Serca2-FL and PLN-KO alleles were bred to homozygosity. These mice were Serca2^fl/fl^;PLN^–/–^ and either expressed (TG) or did not express (NTG) the αMHC-MerCreMer transgene (maintained heterozygous), allowing for Serca2 gene disruption upon tamoxifen injection, as confirmed by genotyping.

In a separate cross, Serca2^fl/fl^;Tg:αMHC-MerCreMer mice were bred with mice expressing a PKA-insensitive mutant of cTnI, cTnI^Ala2^, in which the PKA target serines 23/24 have been mutated to alanines (33) (provided by Richard Moss, University of Wisconsin, Madison, Wisconsin, USA). To ensure the complete expression of the PKA-insensitive transgene, this line was maintained on a background null for endogenous cTnI (cTnI^–/–^) (51), termed cTnI^–/–^;Tg:αMHC-cTnI^Ala2^. Heterozygous siblings were bred to homozygosity to create the line Serca2^fl/fl^;cTnI^–/–^;Tg:αMHC-MerCreMer;Tg:αMHC-cTnI^Ala2^, as confirmed by genotyping.

### Metoprolol survival studies in Serca2a gene–deleted mice

Two randomly assigned groups of SERCA2a^fl/fl^ mice were injected i.p. with tamoxifen to induce excision of the SERCA2a gene by Cre recombinase with subsequent termination of SERCA2a expression. The first group received metoprolol, a selective β1-adrenergic receptor antagonist, at a concentration of 2 mg/mL in drinking water from the time of tamoxifen injection until death. Medicated water was made fresh every 2 days using powdered metoprolol (Sigma-Aldrich) mixed into sterilized water from the same stock of water as the nonmedicated drinking water used for group 2. Medicated water was kept in red UV-filtering bottles to prevent degradation of the drug. Mice were allowed to drink ad libitum, and water was not restricted in either group (treated or control). Mice were euthanized if veterinary services determined that the health of the mouse had decompensated to a point where it was unethical to allow the mouse to live in that state, generally < 12 hours before expected death. Two mice were euthanized in this manner. Results were analyzed in Prism (GraphPad), using a survival function to give Kaplan-Meier survival statistics.

### Western blotting

For Figure 1, hearts were excised from the mice 4 weeks after tamoxifen and frozen quickly in liquid nitrogen. For Figure 4 and Figure 5, hearts went through the Langendorff procedure described above, where they had baseline measurements and ISO measurements; then, they were washed out until they reached pre-ISO function, at which time they were quickly frozen. The hearts were then pulverized and solubilized in RIPA buffer. Brief sonication samples were added to Laemmli buffer plus β-mercaptoethanol and boiled for 5 minutes. Samples were then run on 4%–20% or 12% Bio-Rad Criterion TGX gels. Gels were transferred to PVDF membranes and then blocked overnight at 4°C in 5% milk TBS-T or 3% BSA TBS-T. Primary antibodies were added in blocking buffer and incubated for 1 hour at room temperature. Primary antibodies used were SERCA2 (Abcam, ab150435, 1:1,000), phospho-PLN (MilliporeSigma, 07-052, 1:500), PLN (Invitrogen, MA3-922, 1:1,000), phospho-cTnI S23/S24 (Cell Signaling Technology, 4004, 1:500), and cTnI (Novus, 4C2cc, 1:2,000). Secondary antibodies used were IRDyeCW goat anti-rabbit (LI-COR, 926-32211) and IRDye 680RD goat anti-mouse (LI-COR, 926-68070) at 1:5,000; they were incubated in blocking buffer for 1 hour at room temperature. Blots were visualized on LI-COR Odyssey scanner and quantified in the analysis software. Full blots are shown in the supplemental figures.

### Rigor and reproducibility

#### Statistics.

Data are presented as mean ± SEM. Data were acquired using LabChart 6 software (AD Instruments) and analyzed using Prism 5.02 (GraphPad). Significance was tested by unpaired 2-tailed *t* test or 1- or 2-way ANOVA with Tukey and Bonferroni post hoc test, where appropriate. Significance was set at *P* < 0.05.

#### Study approval.

All experiments were approved by the University of Minnesota IACUC (NIH Animal Welfare Assurance #A3456-01).

#### Data availability.

Values for all data points in graphs are reported in the Supporting Data Values file.

## Author contributions

FIH and BRT are co–first authors, listed alphabetically; they designed and performed experiments, analyzed data, and edited the manuscript. RG and MW performed experiments. JMM directed the study, designed experiments, analyzed data, wrote the manuscript, and was awarded the funding to conduct the study.

## Supplementary Material

Supplemental data

Unedited blot and gel images

Supporting data values

## Version 1 02/08/2024

In-Press Preview

## Version 2 03/22/2024

Electronic publication

## References

[1] Buda V (2022). An up-to-date article regarding particularities of drug treatment in patients with chronic heart failure. J Clin Med.

[2] Heidenreich PA (2022). 2022 AHA/ACC/HFSA guideline for the management of heart failure: executive summary: a report of the American College of Cardiology/American Heart Association joint committee on clinical practice guidelines. Circulation.

[3] Tsao CW (2022). Heart disease and stroke statistics-2022 update: a report from the American Heart Association. Circulation.

[4] Mann DL, Felker GM (2021). Mechanisms and models in heart failure: a translational approach. Circ Res.

[5] La Franca E (2021). Physiopathology and diagnosis of congestive heart failure: consolidated certainties and new perspectives. Curr Probl Cardiol.

[6] Roh J (2022). Heart failure with preserved ejection fraction: heterogeneous syndrome, diverse preclinical models. Circ Res.

[7] Deichl A (2022). Comorbidities in heart failure with preserved ejection fraction. Herz.

[8] Wilcox JE (2020). Heart failure with recovered left ventricular ejection fraction: JACC scientific expert panel. J Am Coll Cardiol.

[9] Kranias EG, Hajjar RJ (2012). Modulation of cardiac contractility by the phospholamban/SERCA2a regulatome. Circ Res.

[10] Hulot JS (2012). Sarcoplasmic reticulum and calcium cycling targeting by gene therapy. Gene Ther.

[11] Heinis FI (2013). Prominent heart organ-level performance deficits in a genetic model of targeted severe and progressive SERCA2 deficiency. PLoS One.

[12] Andersson KB (2009). Mice carrying a conditional Serca2(flox) allele for the generation of Ca(2+) handling-deficient mouse models. Cell Calcium.

[13] Andersson KB (2009). Moderate heart dysfunction in mice with inducible cardiomyocyte-specific excision of the Serca2 gene. J Mol Cell Cardiol.

[14] Hillestad V (2013). Long-term levosimendan treatment improves systolic function and myocardial relaxation in mice with cardiomyocyte-specific disruption of the Serca2 gene. J Appl Physiol (1985).

[15] Swift F (2012). Extreme sarcoplasmic reticulum volume loss and compensatory T-tubule remodeling after Serca2 knockout. Proc Natl Acad Sci U S A.

[16] Edwards AG (2021). Sarcoplasmic reticulum calcium release is required for arrhythmogenesis in the mouse. Front Physiol.

[17] Boardman NT (2014). Impaired left ventricular mechanical and energetic function in mice after cardiomyocyte-specific excision of Serca2. Am J Physiol Heart Circ Physiol.

[18] Land S (2013). Beta-adrenergic stimulation maintains cardiac function in Serca2 knockout mice. Biophys J.

[19] Li L (2012). Sodium accumulation in SERCA knockout-induced heart failure. Biophys J.

[20] Li L (2011). Calcium dynamics in the ventricular myocytes of SERCA2 knockout mice: a modeling study. Biophys J.

[21] Liu XH (2011). Cardiomyocyte-specific disruption of Serca2 in adult mice causes sarco(endo)plasmic reticulum stress and apoptosis. Cell Calcium.

[22] Stokke MK (2010). Reduced SERCA2 abundance decreases the propensity for Ca2+ wave development in ventricular myocytes. Cardiovasc Res.

[23] Louch WE (2010). Sodium accumulation promotes diastolic dysfunction in end-stage heart failure following Serca2 knockout. J Physiol.

[24] Bers DM (1991). Ca regulation in cardiac muscle. Med Sci Sports Exerc.

[25] Francis GS, Tang WH (2003). Pathophysiology of congestive heart failure secondary to congestive and ischemic cardiomyopathy. Cardiovasc Clin.

[26] Bergamasco A (2022). Epidemiology of asymptomatic pre-heart failure: a systematic review. Curr Heart Fail Rep.

[27] Katz AM (1990). Inotropic and lusitropic abnormalities in heart failure. Eur Heart J.

[28] Katz AM (1989). The myocardium in congestive heart failure. Am J Cardiol.

[29] Katz AM (1988). Influence of altered inotropy and lusitropy on ventricular pressure-volume loops. J Am Coll Cardiol.

[30] Katz AM, Smith VE (1984). Relaxation abnormalities. Part I: mechanisms. Hosp Pract (Off Ed).

[31] Yasuda S (2007). Cardiac transgenic and gene transfer strategies converge to support an important role for troponin I in regulating relaxation in cardiac myocytes. Circ Res.

[32] Metzger JM, Westfall MV (2004). Covalent and noncovalent modification of thin filament action: the essential role of troponin in cardiac muscle regulation. Circ Res.

[33] Stelzer JE (2007). Differential roles of cardiac myosin-binding protein C and cardiac troponin I in the myofibrillar force responses to protein kinase A phosphorylation. Circ Res.

[34] Cohn JN, Francis GS (2012). Stage B, a precursor of heart failure, part II. Heart Fail Clin.

[35] Braunwald E (2015). The war against heart failure: the Lancet lecture. Lancet.

[36] Bers DM (2002). Cardiac excitation-contraction coupling. Nature.

[37] Davis J (2008). Designing heart performance by gene transfer. Physiol Rev.

[38] Schiattarella GG (2021). Metabolic inflammation in heart failure with preserved ejection fraction. Cardiovasc Res.

[39] Schiattarella GG (2019). Nitrosative stress drives heart failure with preserved ejection fraction. Nature.

[40] Tong D (2019). Female sex is protective in a preclinical model of heart failure with preserved ejection fraction. Circulation.

[41] Elliott EB (2012). Isolated rabbit working heart function during progressive inhibition of myocardial SERCA activity. Circ Res.

[42] Luo W (1994). Targeted ablation of the phospholamban gene is associated with markedly enhanced myocardial contractility and loss of beta-agonist stimulation. Circ Res.

[43] Francis GS (2014). Inotropes. J Am Coll Cardiol.

[44] Francis GS (2010). ACCF/AHA/ACP/HFSA/ISHLT 2010 clinical competence statement on management of patients with advanced heart failure and cardiac transplant: a report of the ACCF/AHA/ACP task force on clinical competence and training. J Am Coll Cardiol.

[45] Greenberg BH (2012). The Heart Failure Society of America in 2020: a vision for the future. J Card Fail.

[46] Barnabei MS (2015). Severe dystrophic cardiomyopathy caused by the enteroviral protease 2A-mediated C-terminal dystrophin cleavage fragment. Sci Transl Med.

[47] Wang W (2013). Noncanonical EF-hand motif strategically delays Ca2+ buffering to enhance cardiac performance. Nat Med.

[48] Barnabei MS, Metzger JM (2012). Ex vivo stretch reveals altered mechanical properties of isolated dystrophin-deficient hearts. PLoS One.

[49] Barnabei MS (2010). Influence of genetic background on ex vivo and in vivo cardiac function in several commonly used inbred mouse strains. Physiol Genomics.

[50] Palpant NJ (2009). Single histidine button in cardiac troponin I sustains heart performance in response to severe hypercapnic respiratory acidosis in vivo. FASEB J.

[51] Huang X (1999). Cardiac troponin I gene knockout: a mouse model of myocardial troponin I deficiency. Circ Res.

