# Supplementary material for: Myofilament-based physiological regulatory compensation preserves diastolic function in failing hearts with severe Ca2+ handling deficits
Source: JCI Insight. 2024 Feb 8;9(6):e163334. doi: 10.1172/jci.insight.163334 (PMC11063947; doi:10.1172/jci.insight.163334)
Supplement: Supplemental data [file jciinsight-9-163334-s178.pdf]

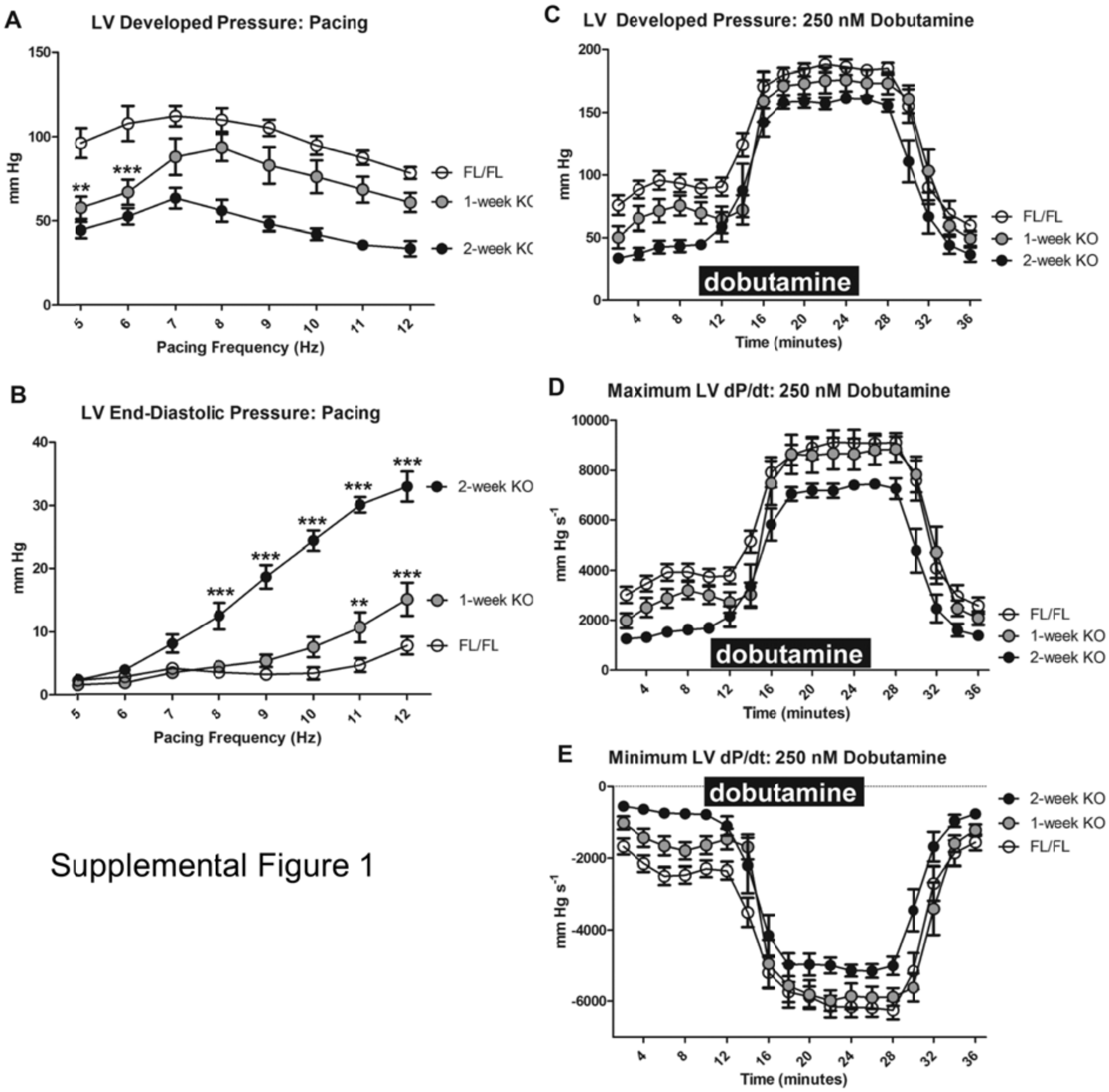

Supplemental Figure 1

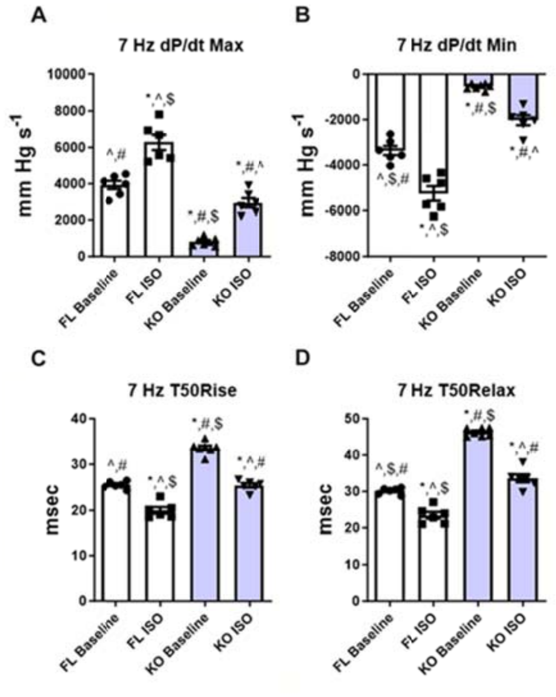

Supplemental Figure 2

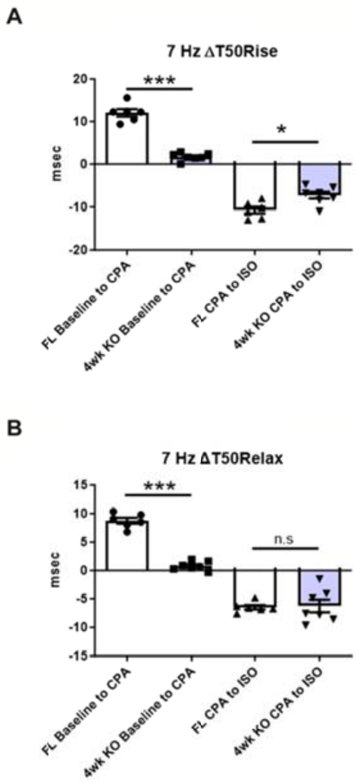

Supplemental Figure 3

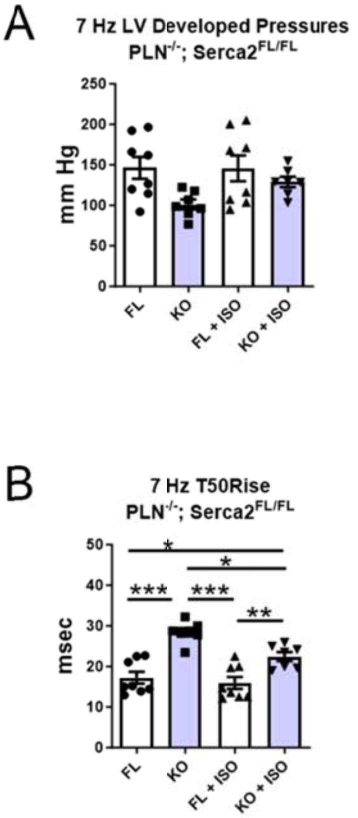

Supplemental Figure 4
