# Supplementary figures and images for: Myofilament-based physiological regulatory compensation preserves diastolic function in failing hearts with severe Ca2+ handling deficits
Source: JCI Insight. 2024 Feb 8;9(6):e163334. doi: 10.1172/jci.insight.163334 (PMC11063947; doi:10.1172/jci.insight.163334)

Figure 1 full blots

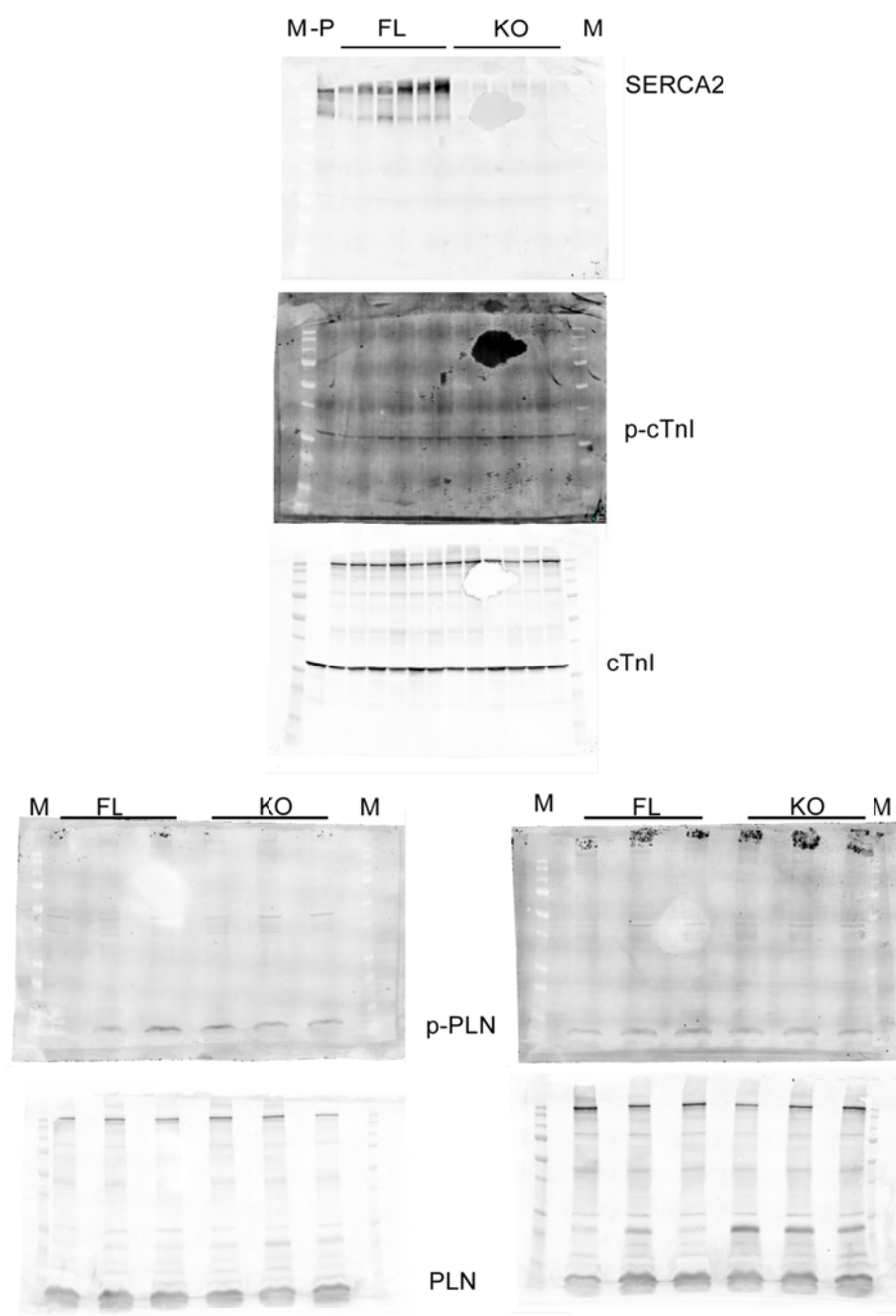

Figure 4 full blots

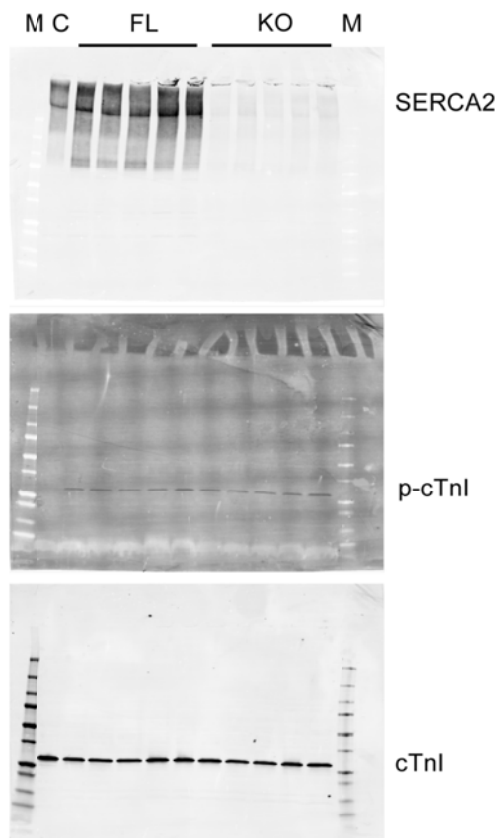

Figure 5 full blots

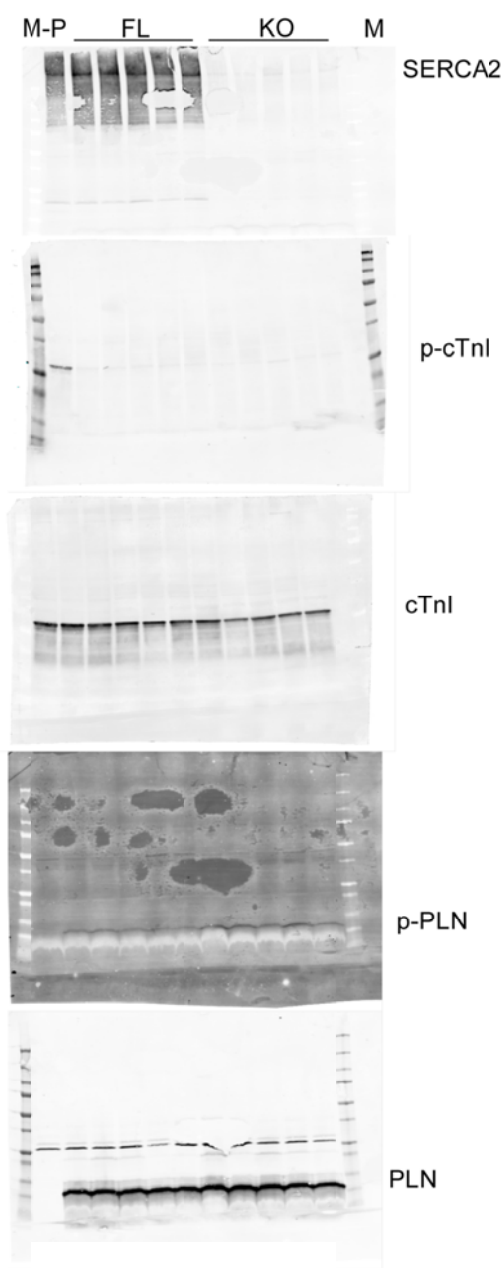

Supplement: Unedited blot and gel images [file jciinsight-9-163334-s179.pdf]
